# Comparative Analysis of the Fibroblastic Response and Collagen and Elastic Fiber Formation After Implantation of Polypropylene Versus an Electrospun Biodegradable Polymer for Reinforcement of the Musculo-Fascial Wall

**DOI:** 10.3390/jfb17100496

**Published:** 2026-10-01

**Authors:** Igor A. Eisenach, Elena L. Lushnikova, Galina A. Lapii, Alexey V. Kuznetsov, Larisa Y. Kostina

**Affiliations:** 1 Institute of Molecular Pathology and Pathomorphology, Federal Research Center for Fundamental and Translational Medicine, 2 Timakova St., Novosibirsk 630117, Russia; ellushnikova@frcftm.ru (E.L.L.); galapii@frcftm.ru (G.A.L.); 2 Federal State Budgetary Educational Institution of Higher Education, Novosibirsk State Medical University, Ministry of Health of Russia, 52 Krasniy Prospekt, Novosibirsk 630091, Russia; 1xo2788353@mail.ru (A.V.K.); klory@mail.ru (L.Y.K.)

**Keywords:** biodegradable polymer, polypropylene, implant, the musculo-fascial “corset”, connective tissue, cellular and extracellular components, collagen type I and III, elastic fibers

## Abstract

The replacement of polypropylene with biodegradable polymers for surgical reinforcement of musculo-fascial structures is of considerable clinical interest. A study in 98 laboratory rats with implanted polypropylene or biodegradable biopolymer demonstrated the formation of dense irregular connective tissue at the surgical site instead of biopolymer and dense regular connective tissue around the polypropylene. The fibroblastic response to the biopolymer lagged behind that to polypropylene by approximately nine months, reflecting gradual polymer degradation, but did not differ between groups at 12 months, when the biopolymer had completely biotransformed. In both groups, newly formed connective tissue contained numerous elastic fibers and relatively fewer type I (COLI) and type III (COLIII) collagen fibers compared with intact preoperative tissue. In peri-implant tissue from both groups, COLIII fibers significantly predominated over COLI, with a higher volume fraction of COLIII around the polypropylene than around the biopolymer, whereas COLI was significantly more abundant in intact tissues. These findings are consistent with the good extensibility and low tear resistance of newly formed connective tissue. In the polypropylene group, polymer filaments reinforced this tissue mechanically. Further analysis of explanted tissues is required to determine the functional properties of connective tissue remodeled at the site of a degraded biopolymer implant.

## 1. Introduction

The failure of the musculo-fascial “corset” is common in the general population and manifests in several clinical forms. In different age groups, hernias of the anterior abdominal wall occur in 3.2–19.7% of cases, and pelvic organ prolapse with concomitant stress urinary incontinence affects up to 70% of women [1,2,3,4,5,6,7]. One of the leading causes of these conditions is thought to be impaired connective tissue formation, particularly in collagen and elastic fibers and their relative proportions [8,9].

Cellular components, especially fibroblasts and fibrocytes, also determine the functional properties of connective tissue [10,11]. Fibroblasts are the primary cells responsible for synthesizing these fibers. Type I collagen provides tensile strength and resistance of connective tissue to stretching and tearing, whereas type III collagen supports the framework of blood vessels and hollow organs, contributing to tissue elasticity and extensibility [9,12]. Functionally, type III collagen occupies an intermediate position between type I collagen and elastic fibers. Elastic fibers themselves determine the capacity of tissues to stretch and restore their original shape, thereby imparting elasticity [10,13,14].

Although medicine has not yet resolved the problem of genetically correcting collagen and elastic fiber synthesis in humans, surgeons can correct the clinical manifestations of these disorders surgically [1,15]. Techniques that rely solely on autologous tissue are associated with high recurrence rates [1,16]. Over recent decades, polypropylene and other synthetic polymer implants have been widely used to reinforce the musculo-fascial corset, and mesh-based procedures are now considered the gold standard for repairing hernias of the anterior abdominal wall [2,3]. By contrast, the use of polypropylene implants for pelvic organ prolapse has been less successful because of high complication rates, limiting their application in this setting [4,5,6]. The use of permanent synthetic implants has also been questioned in the context of the recently described autoimmune/inflammatory syndrome induced by adjuvants (ASIA), as lifelong implanted materials, even highly bioinert ones, may trigger a systemic immune response [17,18,19,20].

Biodegradable polymers that gradually “dissolve” within tissues are therefore of considerable interest for correcting musculo-fascial defects [21,22]. In the early postoperative period, a biopolymer implant can provide mechanical support to a previously prolapsed musculo-fascial wall [23]. In the late postoperative period, approximately 1–3 months after surgery, ongoing biotransformation of the polymer leads to the formation of connective tissue at the implant site [24,25]. The extent to which this newly formed connective tissue achieves sufficient strength and can fully function as a musculo-fascial corset is of practical importance [26,27]. To a large extent, its cellular composition, the qualitative profile of collagen and elastic fibers, and their ratio determine the functional properties of the new tissue [12,23,28].

More informative data can be obtained by comparing experimental biopolymers with widely used surgical materials such as polypropylene mesh. The aim of this study was to perform a comparative morphometric and immunohistochemical analysis of the cellular composition and qualitative characteristics of collagen and elastic fibers at the implantation site of polypropylene and an electrospun biodegradable polymer for reinforcement of the musculo-fascial wall.

## 2. Materials and Methods

Three groups of male Wistar rats (3 months of age) were used: Group 1 (*n* = 56), Group 2 (*n* = 21), and Group 3 (*n* = 21). All animals underwent subcutaneous implantation of polymeric materials in the dorsal region. The implant was installed in the interfascial space, between the superficial fascia and the fascia propria of the back muscles. In Group 1, a 1 × 1 × 0.2 cm polypropylene mesh (specific weight 28 g/m^2^) was implanted on the left side of the spine, and a 1 × 1 × 0.2 cm biopolymer matrix composed of 65% polycaprolactone (PCL) and 35% polytrimethylene carbonate (PTMC) was implanted on the right side. In Group 2, a 1 × 1 × 0.2 cm polypropylene mesh (28 g/m^2^) was implanted on the left side of the spine. In Group 3, a 1 × 1 × 0.2 cm PCL/PTMC biopolymer matrix (65/35%) was implanted on the left side of the spine. The polypropylene mesh used in the study is routinely employed in surgical correction of musculo-fascial wall failure (THT Bio-Science, Montpellier, France). The biopolymer matrix was produced experimentally by electrospinning. A solution of PCL and PTMC (Sigma-Aldrich, St. Louis, MO, USA) in chloroform was prepared and electrospun using an MECC electrospinning unit (MECC Co., Ltd., Fukuoka, Japan), yielding a nonwoven PCL/PTMC scaffold. The local protocol was approved by the Ethics Committee of The Federal Research Center Institute of Cytology and Genetics Siberian Branch of the Russian Academy of Sciences (protocol code: 156/1; date of approval: 18 September 2023).

### 2.1. Tissue Analysis, Cellular Components (Morphometry)

In Group 1 (*n* = 56), 8 rats were euthanized at each time point: 2 weeks and 1, 2, 3, 6, 9, and 12 months after implantation. Histological samples were collected as tissue blocks containing the polypropylene or biopolymer implant together with the surrounding tissue. After fixation in 10% neutral-buffered formalin and standard tissue processing in a Leica TP1020 automatic tissue processor (Leica Biosystems, Nussloch, Germany), specimens were embedded in paraffin blocks. Sections 3–4 μm thick were cut on a Leica RM2235 rotary microtome (Leica Biosystems, Nussloch, Germany). Paraffin sections were stained with hematoxylin and eosin using a Leica ST5010 stainer (Leica Biosystems, Nussloch, Germany) and with van Gieson stain to visualize collagen fibers. Slides were examined with an Olympus CX31 microscope equipped with a Nikon DS-Fi1 digital camera (Tokyo, Japan). In the implantation zones, granulomatous tissue in close proximity to the synthetic material was evaluated, and the qualitative and quantitative characteristics of cellular and extracellular components of the surrounding connective tissue were assessed. The numbers of fibrocytes and fibroblasts were determined by counting at least 100 cells per field of view at 400× magnification and expressing each cell type as a percentage of the total. For each animal, ten visual fields were analyzed at two or three sections of the peri-implantation tissue, depending on their quality. One section was always taken from the central implantation zone and the second at the maximum distance in the peripheral zone. If the size of the explanted material allowed, the third section was taken from the peripheral zone on the opposite side and at the maximum distance from the second section. For each animal, a simple arithmetic mean was calculated and used as the observation unit. Thus, at each time point, two groups with different implanted materials were compared with the same number of observations (*n* = 8). Quantitative measurements were obtained automatically using BioVision 4.0 image analysis software. Mean fibrocyte and fibroblast counts at different time points were compared using the nonparametric Mann–Whitney U test and different materials were compared using the nonparametric Wilcoxon T test (Wilcoxon signed-rank test), implemented in Statistica 13 and Python 3.14 (libraries: Pandas 2.3.3, Seaborn 0.13.2, NumPy 2.3.0).

### 2.2. Tissue Analysis, Extracellular Components: Collagen and Elastic Fiber Formation (Immunohistochemistry and Morphometry)

In Group 2 (polypropylene; *n* = 21) and Group 3 (biopolymer; *n* = 21), a small skin sample at the site of future implantation was obtained from each animal at the time of initial surgery. Tissues representing the deep layers of the dermis and superficial fascia, and adjacent to the fascia propria of the back muscles, were for baseline immunohistochemical and morphometric analysis. All animals in these groups were euthanized 3 months after implantation. Histological specimens were collected as tissue blocks containing the polypropylene implant (Group 2) or biopolymer matrix (Group 3) together with the surrounding tissue. After fixation in 10% neutral-buffered formalin and standard tissue processing in a Leica TP1020 automatic tissue processor (Leica Biosystems, Nussloch, Germany), specimens were embedded in paraffin blocks. Sections 3–4 μm thick were prepared using a Leica RM2235 rotary microtome (Leica Biosystems, Nussloch, Germany). 129 Paraffin sections were stained with hematoxylin and eosin on a Leica ST5010 stainer (Leica Biosystems, Nussloch, Germany) and with van Gieson stain to visualize collagen fibers. Histological preparations were examined with an Olympus CX31 microscope equipped with a Nikon DS-Fi1 digital video camera (Tokyo, Japan). The immunohistochemical procedure, adapted for this laboratory, was performed in accordance with established guidelines for immunohistochemical studies and the antibody manufacturers’ recommendations [29,30]. Before staining, sections were deparaffinized and subjected to heat-induced antigen retrieval in a PT Link module (Dako, Glostrup, Denmark) using citrate buffer (pH 6.0) at 95 °C for 60 min. Endogenous peroxidase activity was blocked with 3% H_2_O_2_, followed by protein blocking with serum. Tissue sections were then incubated with rabbit polyclonal antibodies against COL3A1 and COL1A1 (Elabscience, Wuhan, China). All antibodies were diluted and applied according to the manufacturers’ instructions. A peroxidase-labeled polymer detection system was used for immunostaining, and cell nuclei were counterstained with hematoxylin. Immunohistochemical results were evaluated semi-quantitatively. For each antibody, numerical density, defined as the proportion of positively stained cellular structures, was determined. For COL3A1 and COL1A1, positivity was assessed by the presence of specific cytoplasmic staining. In the implant area, granulomatous tissue was evaluated, and blood vessels were counted along the section surface at 200× magnification. The volume density of elastic fibers and of structures positively stained for COL3A1 and COL1A1 was assessed in 10 fields of view at 400× magnification using a morphometric grid with 121 nodes. Volume density (Vv) was calculated according to the formula Vv = x/n, where x is the number of grid points overlying the structure of interest and n is the total number of points counted [31].

Mean volume densities of elastic fibers and COL3A1- and COL1A1-positive structures in different Groups (2, 3) were compared using the nonparametric Mann–Whitney U test and those within a single Group were compared using the nonparametric Wilcoxon T test. Tissue parameters were compared between baseline (intact tissues) and 3 months after implantation for Group 2 (polypropylene) and Group 3 (biopolymer). Baseline values for elastic fibers, COL3A1, and COL1A1 were calculated using data from all animals in Groups 2 and 3 combined (*n* = 42), because the intact tissue values for both groups with future implantation were almost equal, and also to simplify the presentation of the results in graphical form, whereas 3-month values were calculated separately for each group (*n* = 21). When comparing mean values for intact tissues with those in the comparison groups, statistical significance was assessed using Wilcoxon T test. Statistical analysis and graphical presentation of the data were performed using Statistica 13 and Python 3.14 (libraries: Pandas 2.3.3, Seaborn 0.13.2, NumPy 2.3.0).

## 3. Results

### 3.1. Tissue Analysis of Cellular Components (Morphometry)

No complications were observed in Group 1 animals throughout the 12-month experimental period. In Group 1, macroscopic examination of specimens containing the biopolymer showed no visible capsule thickening over time. Distinct matrix boundaries were evident for up to 6 months after implantation. At 9 months, a uniform cellular matrix structure was observed, with ulcerated edges and areas of degradation, while the overall dimensions of the material remained unchanged. At 12 months, the biopolymer margins were no longer discernible, and a dense band of connective tissue with small white islands of residual, nondegraded material was visible at the implantation site.

After polypropylene implantation in Group 1, macroscopic specimens showed the formation of an additional layer of connective tissue reinforced by polypropylene filaments around the implant. Examination of the excised tissue surrounding the polypropylene implant revealed a white connective tissue capsule forming around the material as early as 2 weeks after surgery, which subsequently thickened over time.

**Figure 1:**
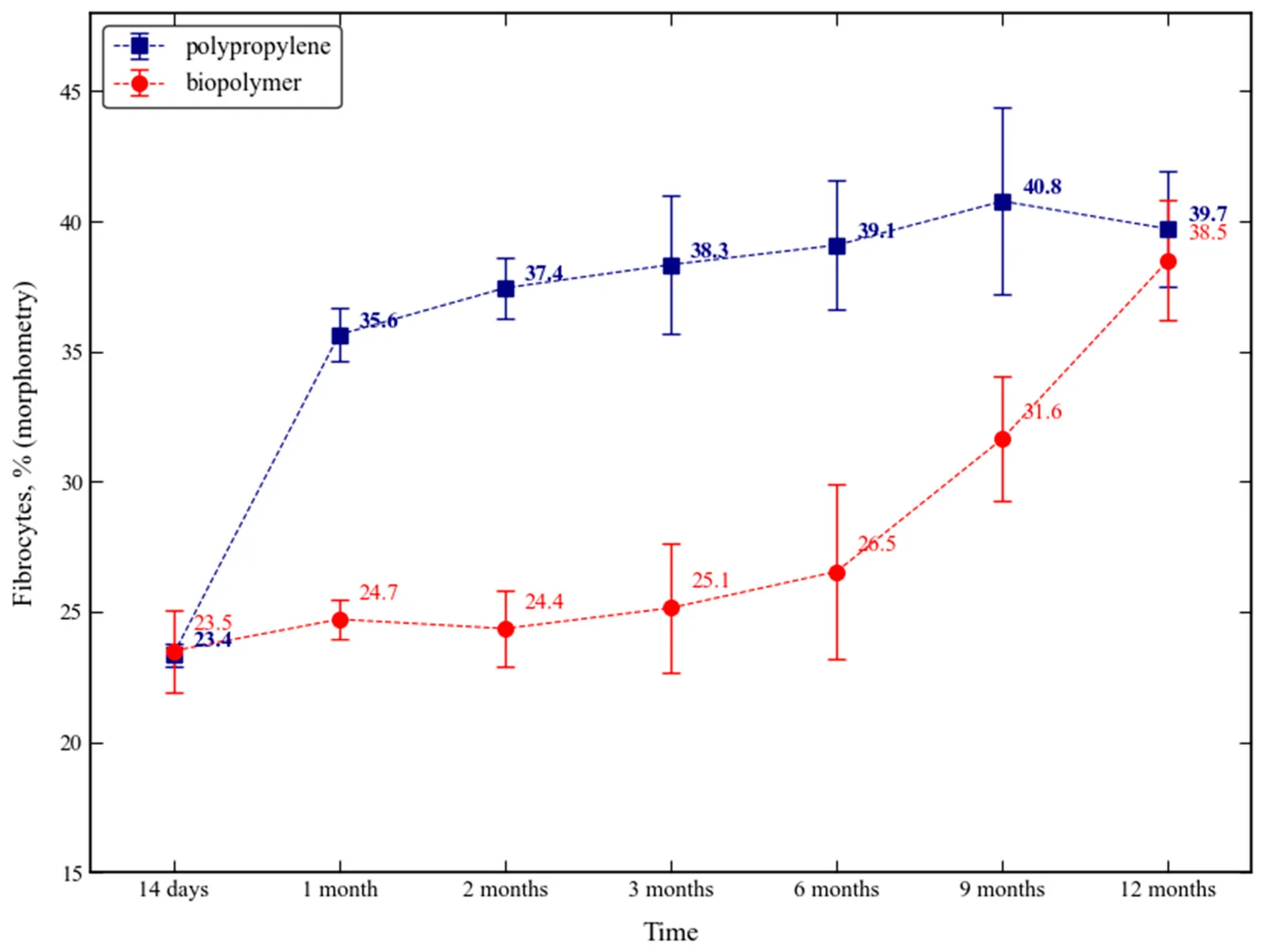
Dynamics of fibrocyte reaction (mean ± SD) to implanted polypropylene (blue line) and biopolymer (red line) over time (morphometry (%) in tissues, Group 1).

**Figure 2:**
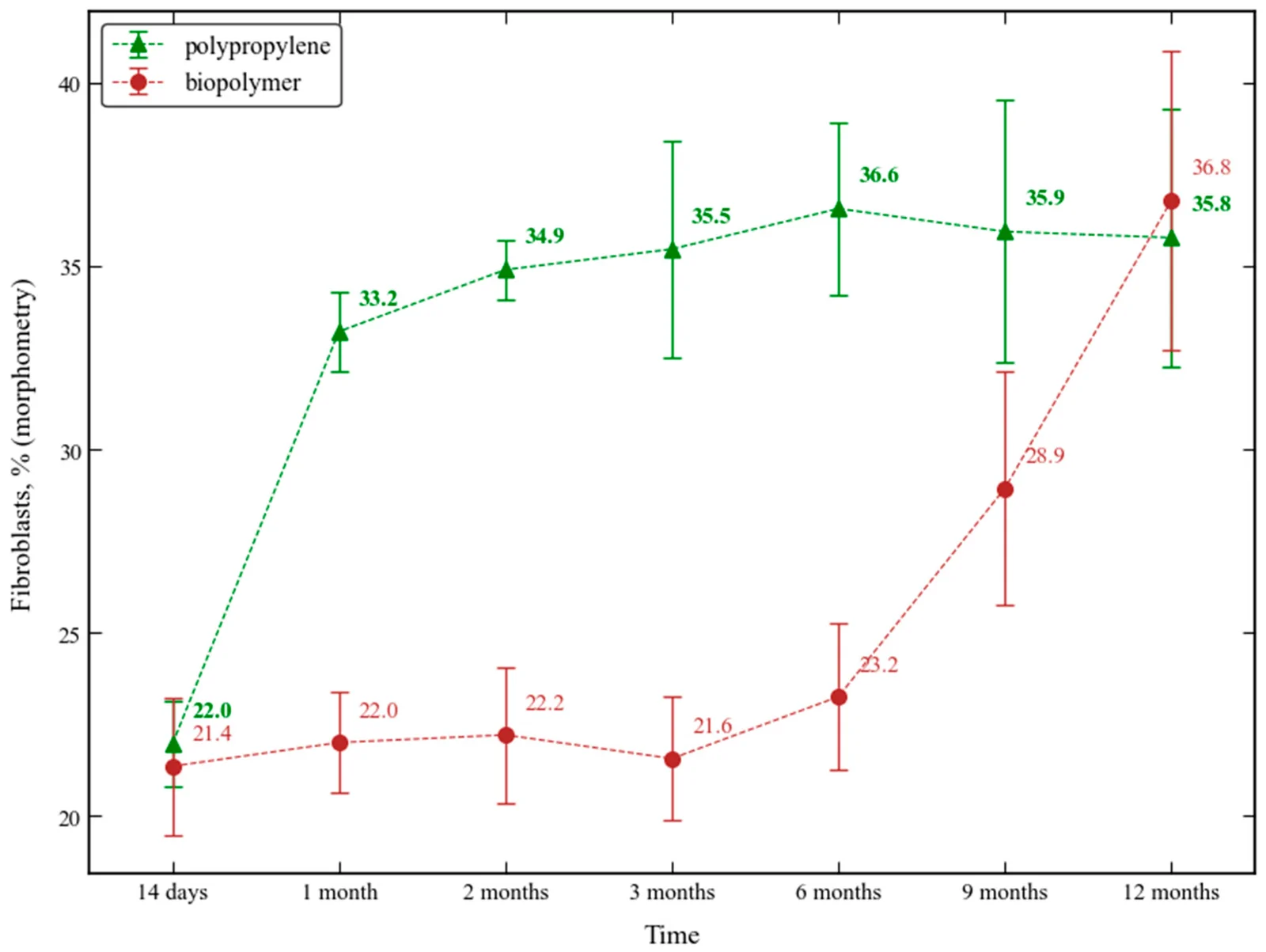
Dynamics of fibroblast reaction (mean ± SD) to implanted polypropylene (green line) and biopolymer (burgundy line) over time (morphometry (%) in tissues, Group 1).

After 2 weeks, microscopic examination revealed a zone of cellular infiltration around both synthetic materials. Around the polypropylene, cells accumulated along each individual fiber of the implant. Around the biopolymer, given its nonfibrous matrix structure and physical properties, cellular infiltration was detected only along the matrix perimeter, with no migration of cells into the material itself. During this period, the infiltrate consisted of both resident and recruited (nonresident) inflammatory cells. Leukocytes, macrophages, and lymphocytes that had migrated from local vessels into the altered zone were abundant with both materials. Two weeks after surgery, cells involved in connective tissue formation—fibroblasts and fibrocytes—were already present in substantial numbers. Collagen fibers and blood vessels were not yet clearly distinguishable. At this time point, the mean fibrocyte and fibroblast counts within the infiltrate did not differ significantly between the polypropylene and biopolymer implants (fibrocytes: T = 13, *p* = 0.87; fibroblasts: T = 15, *p* = 0.67). Figure 1 shows the dynamic of the mean for fibrocytes and Figure 2 that of for fibroblasts.

After 1 month, the cellular infiltrate around the biopolymer was still confined mainly to the matrix perimeter, and the first signs of cell migration into the material were observed. The infiltrate showed a layered organization: the inner layer, adjacent to the synthetic material, contained numerous leukocytes and lymphocytes; more superficially, scattered foreign body-type giant cells were present, and beyond them fibroblasts and fibrocytes were visible.

During this period, a circumferential ridge of granulation tissue had already formed around the polypropylene mesh fibers. Fibroblasts were evident within the infiltrate around the polypropylene fibers, sometimes spreading in a thin layer parallel to the implant surface. At the same time, fibroblasts had begun to synthesize collagen fibers, which were arranged circumferentially around the polypropylene fibers, forming ring-like structures along their surface. Collagen fibers were also observed around the biopolymer; however, in contrast to the polypropylene samples, these fibers were distributed more uniformly across the entire matrix surface.

The cellular infiltrate around the biopolymer contained significantly fewer fibroblasts and fibrocytes than the infiltrate around the polypropylene implant (*p* < 0.05).

In later stages, qualitative and gross morphologic changes in the infiltrate surrounding the polypropylene implant were minimal. In contrast, the inflammatory infiltrate around the biopolymer progressively extended deeper into the matrix over time, although no obvious qualitative changes in the inner and outer layers were observed.

From the second month through the twelfth month, fibrocyte and fibroblast counts within the infiltrate remained significantly higher around the polypropylene than around the biopolymer (*p* < 0.05). At 12 months, however, fibrocyte and fibroblast counts no longer differed significantly between the two materials (fibrocytes: T = 9, *p* = 0.20; fibroblasts: T = 11, *p* = 0.60). The temporal pattern of fibrocyte counts in the infiltrate around the polypropylene was as follows. A significant increase in fibrocyte number was observed at 1 month compared with 2 weeks (U = 0, *p* < 0.05) and at 2 months compared with 1 month (U = 8.5, *p* < 0.05). The increase in fibrocyte counts at 3 months compared with 2 months was small and not statistically significant (U = 30, *p* = 0.87). Thereafter, fluctuations in fibrocyte counts up to 12 months were not statistically significant (U = 26, *p* = 0.56). The temporal pattern of fibroblast counts within the infiltrate around the polypropylene implant was as follows. A significant increase in fibroblast number, paralleling the increase in fibrocytes, was observed at 1 month compared with 2 weeks (U = 0, *p* < 0.05) and at 2 months compared with 1 month (U = 5.5, *p* < 0.05). The increase in fibroblast counts at 3 months compared with 2 months was small and not statistically significant (U = 29.5, *p* = 0.83). In subsequent months, increases and decreases in fibroblast counts, including at 9 and 12 months, were not statistically significant (U = 30.5, *p* = 0.92).

The temporal pattern of fibrocyte counts in the infiltrate around the biopolymer was as follows. During the first 6 months, no significant increase in fibrocyte number was detected (*p* > 0.05). A significant increase in fibrocyte counts was observed at 9 months compared with 6 months (U = 6, *p* < 0.05) and at 12 months compared with 9 months (U = 0, *p* < 0.05).

The temporal pattern of fibroblast counts in the infiltrate around the biopolymer was similar but shifted to later time points. The increase in fibroblast counts at 1 month compared with 2 weeks (U = 25.5, *p* = 0.53) and at 2 months compared with 1 month (U = 30.5, *p* = 0.92) was small and not statistically significant. No significant changes in fibroblast counts were observed at 3 and 6 months (*p* > 0.05). By 9 months, fibroblast counts had increased significantly compared with 6 months (U = 2, *p* < 0.05), and a further significant increase was noted at 12 months compared with 9 months (U = 4, *p* < 0.05). The results of morphometry are presented in Table 1.

### 3.2. Tissue Analysis, Extracellular Components: Collagen and Elastic Fiber Formation (Immunohistochemistry and Morphometry)

**Figure 3:**
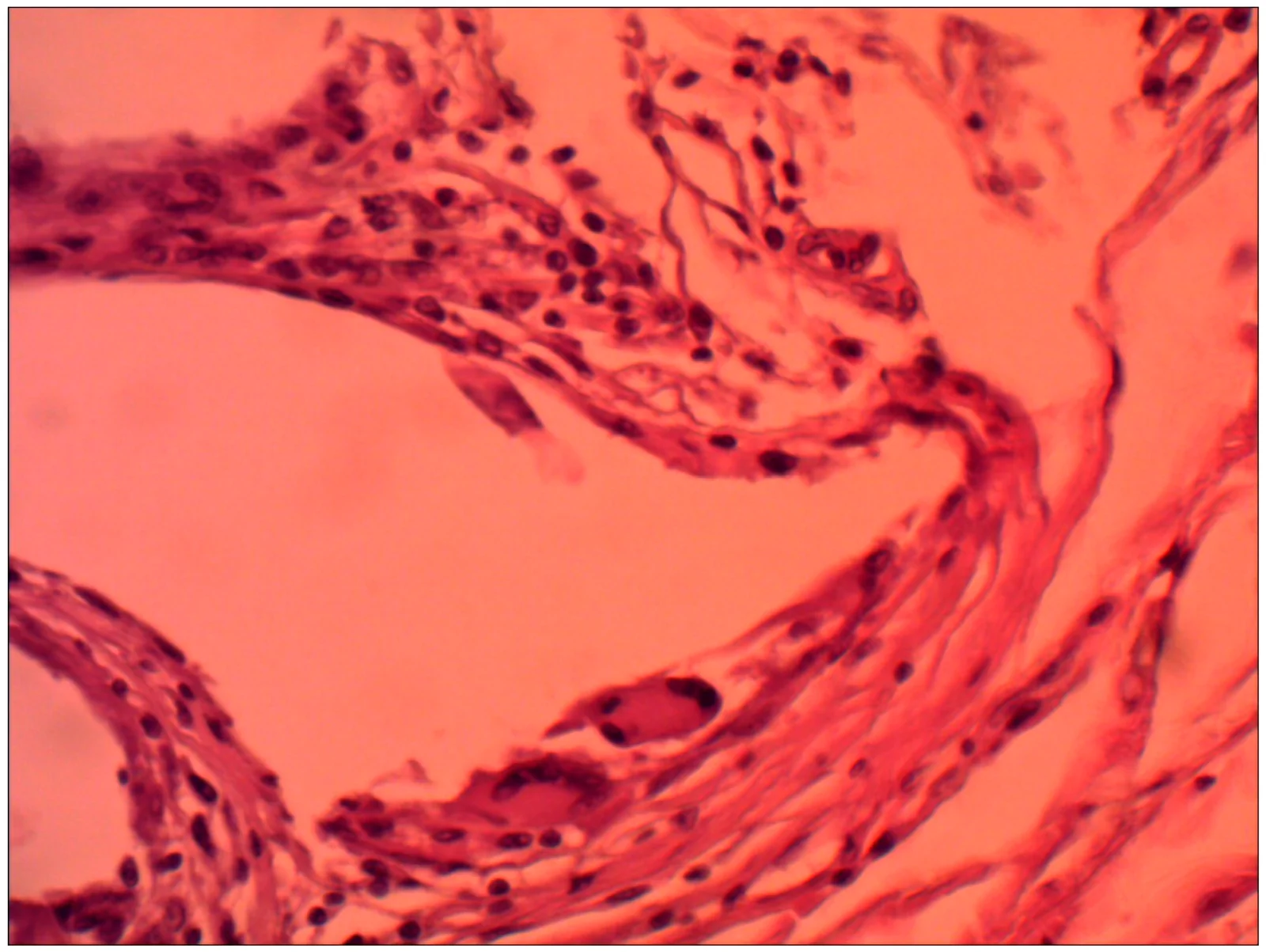
Group 2 with polypropylene, after 3 months of the experiment. Collagen and elastic fibers were oriented predominantly around the polypropylene filaments. Hematoxylin and eosin staining. Magnification 200.

**Figure 4:**
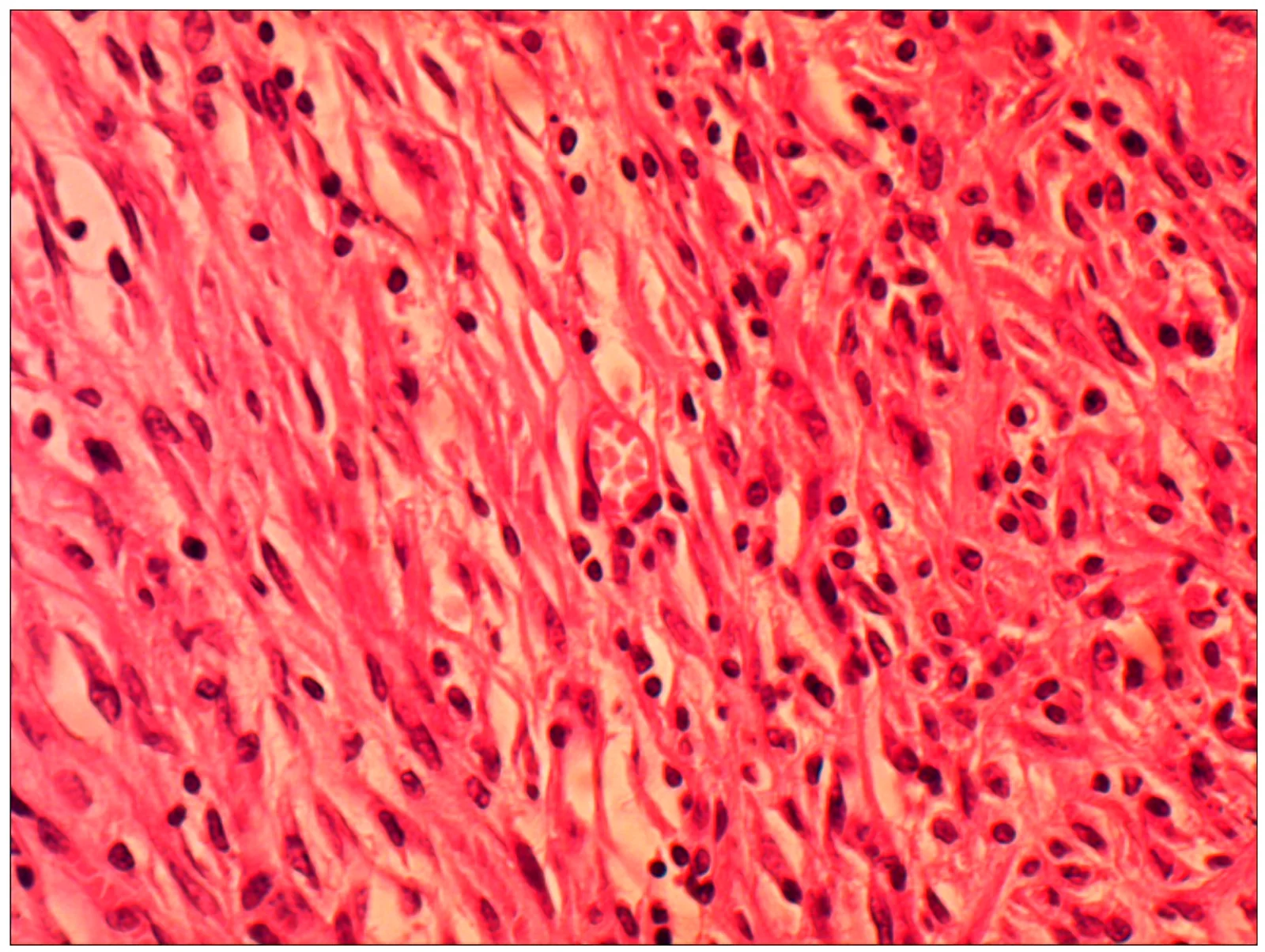
Group 3 with biopolymer, after 3 months of the experiment. Collagen and elastic fibers were distributed uniformly and diffusely. Hematoxylin and eosin staining. Magnification 200.

No complications were observed in either Group 2 or Group 3 animals throughout the experimental period. Three months after implantation, macroscopic examination revealed no external signs of inflammation around either implant type. Connective tissue uniformly encapsulated the implants in both groups, and active connective tissue growth with formation of collagen and elastic fibers was evident around the polymers, as can be seen in Figure 3 and Figure 4.

**Figure 5:**
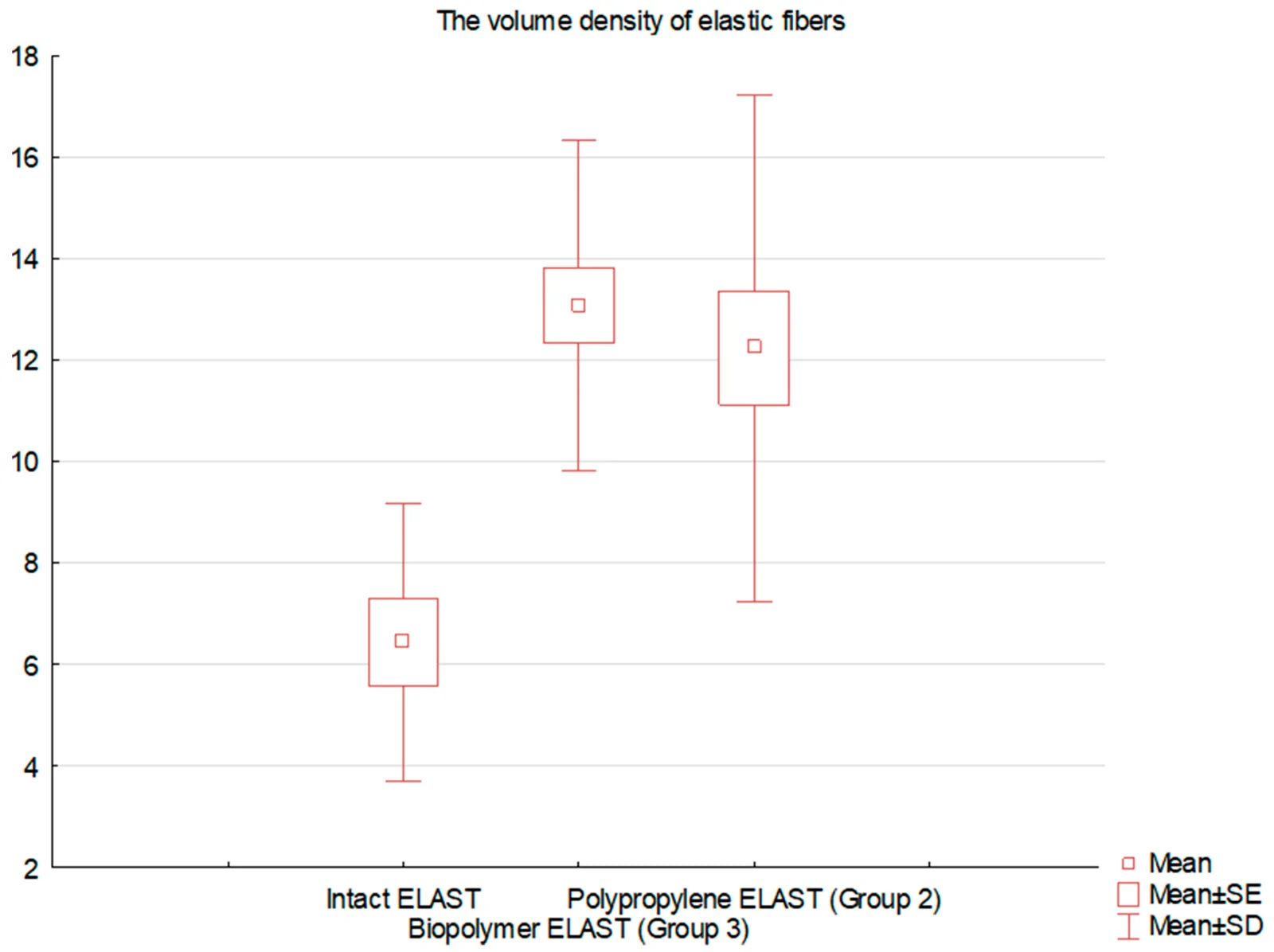
The volume density of elastic fibers in the comparison group, from left to right: in intact tissue; in Group 3 with the biopolymer; in Group 2 with the polypropylene (3 months after implantation).

In intact tissues prior to polymer implantation, the volume density of elastic fibers was ELAST 6.4 ± 2.7, as can be seen in Figure 5.

**Figure 6:**
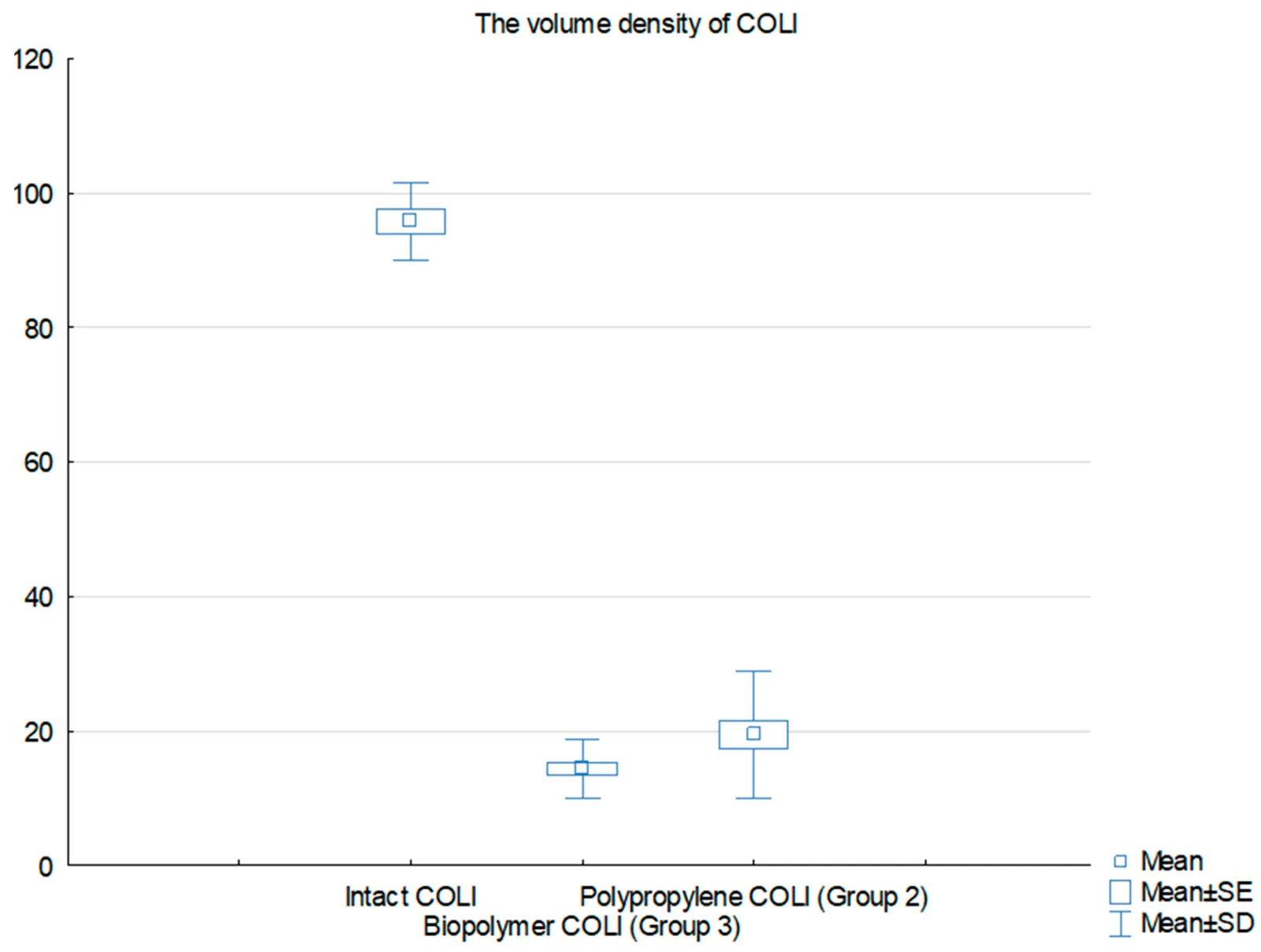
The volume density of collagen type I in the comparison group, from left to right: in intact tissue; in Group 3 with the biopolymer; in Group 2 with the polypropylene (3 months after implantation).

**Figure 7:**
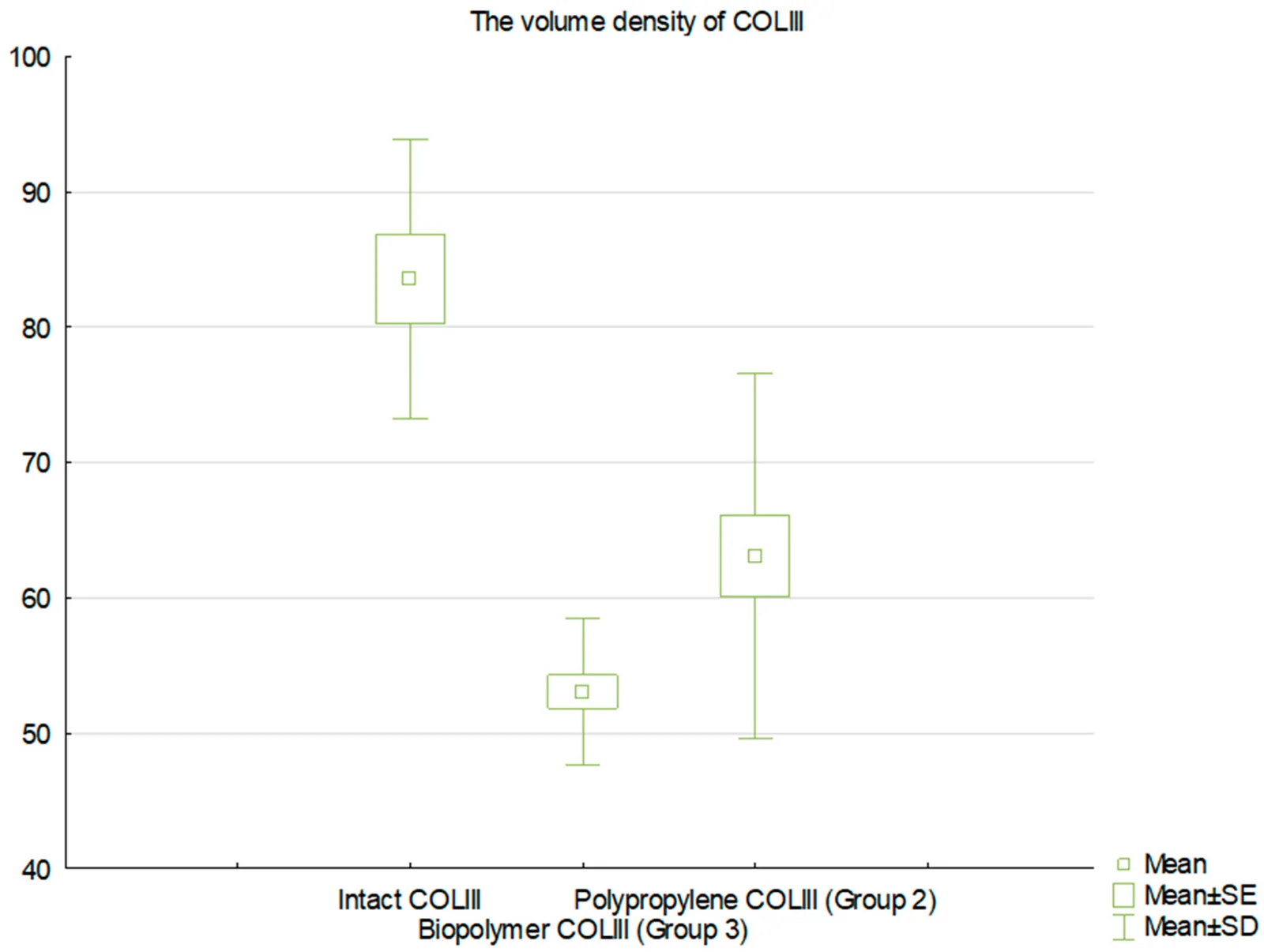
The volume density of collagen type III in the comparison group, from left to right: in intact tissue; in Group 3 with the biopolymer; in Group 2 with the polypropylene (3 months after implantation).

The corresponding values for collagen fibers were COLI 95.7 ± 5.8 and COLIII 83.5 ± 10.4, as can be seen in Figure 6 and Figure 7.

Tissues contained significantly more COLI than COLIII collagen (U = 0, *p* < 0.05), and the COLI/COLIII ratio was 0.87. Elastic fibers were relatively sparse and markedly less abundant than collagen fibers; the ratio of total collagen (COLI + COLIII) to elastic fibers, (COLI + COLIII)/ELAST, was 28 as can be seen in Table 2.

On this basis, the extracellular matrix of intact tissues was dominated by collagen fibers with a predominance of type I collagen.

In Group 2, 3 months after polypropylene implantation, the volume density of elastic fibers in peri-implant tissue was ELAST 12.2 ± 5. The volume densities of collagen fibers were COLI 19.4 ± 9.5 and COLIII 63.1 ± 13.5. COLIII collagen was significantly more abundant than COLI (*p* < 0.05), and the COLIII/COLI ratio was 3.25. Elastic fibers in peri-implant tissue around polypropylene were significantly more numerous than in intact tissue (*p* < 0.05) but remained significantly less abundant than collagen fibers. The ratio (COLI + COLIII)/ELAS was 6.76.

In Group 3, 3 months after biopolymer implantation, the volume density of elastic fibers in peri-implant tissue was ELAST 13.1 ± 3.3. The corresponding values for collagen fibers were COLI 14.3 ± 4.4 and COLIII 53.1 ± 5.5. COLIII collagen was several-fold more abundant than COLI (*p* < 0.05), and the COLIII/COLI ratio was 3.71. As in the polypropylene group, elastic fibers in peri-implant tissue around the biopolymer were significantly more numerous than in intact tissue (*p* < 0.05), yet still several-fold less abundant than collagen fibers; here, (COLI + COLIII)/ELAST was 5.15.

Overall, after implantation of synthetic materials, tissues in both groups (Group 2 and Group 3) contained significantly more elastic fibers than intact tissues (*p* < 0.05), whereas the two implant groups with polypropylene (Group 2) and the biopolymer (Group 3) did not differ significantly from each other in elastic fiber density (U = 155.5, *p* = 0.23).

In both groups, the volume densities of COLI and COLIII collagen were significantly lower than in intact tissues (*p* < 0.05). Moreover, in both peri-implant groups, in contrast to intact tissue, COLIII significantly predominated over COLI (*p* < 0.05). For COLI volume density, the polypropylene and biopolymer groups did not differ significantly (U = 126.5, *p* = 0.051). By contrast, the volume density of COLIII collagen was significantly higher in the polypropylene group (Group 2) than in the biopolymer group (Group 3; U = 66, *p* < 0.05).

## 4. Discussion

Morphometric findings indicate that the initial tissue response, characterized by fibrocyte and fibroblast migration, was independent of implant type: during the first 2 weeks, the numbers of these cells in the infiltrates did not differ significantly between the materials. This similarity suggests that, given an equivalent early response to different implants, the connective tissue formed shortly after surgery might not exhibit major qualitative or quantitative differences. Subsequently, from 1 to 9 months, the fibroblastic response to the biopolymer lagged behind that to polypropylene, reflecting ongoing polymer biotransformation. During this period, the inflammatory infiltrate advanced progressively into the biopolymer matrix, and the influx of leukocytes, lymphocytes, and macrophages diluted the relative proportion of fibrocytes and fibroblasts in Group 3, resulting in lower fibroblast and fibrocyte counts throughout most of the experiment. Over time, however, fibroblast and fibrocyte numbers in the infiltrate around the biopolymer gradually increased, and by 12 months they no longer differed significantly from those in tissue surrounding the polypropylene implant.

In contrast, fibroblast and fibrocyte counts around polypropylene did not change significantly after the second month (*p* > 0.05), suggesting that by 3 months a young connective tissue had already formed at the site of the infiltrate around the polypropylene filaments, with only limited further change at later time points. This interpretation is supported by morphometric data showing that cell counts had already begun to decline at 9 and 12 months, without statistically significant differences between these later time points (*p* > 0.05). These observations imply that, for polypropylene implants, a 3-month follow-up is sufficient to characterize the cellular and extracellular components of newly formed peri-implant connective tissue. They are also consistent with clinical data indicating relatively rapid recovery and early return to normal activity in patients undergoing hernia repair with polypropylene mesh.

At 12 months, as at 2 weeks postoperatively, peri-implant tissues around polypropylene and the biopolymer did not differ significantly in fibrocyte or fibroblast counts. These results support the conclusion that early and late tissue responses converge and are broadly similar regardless of the chemical and physical properties of the implanted material under the conditions of this experiment. The tissue response to a foreign body is uniform, regardless of its physical properties [1,2]. Morphometric studies after 12 months were difficult due to the difficulties in determining the implantation site after the biopolymer matrix had degraded.

According to the immunohistochemical data, stromal structures in intact tissue were dominated by collagen fibers, which were almost 30-fold more abundant than elastic fibers. COLI collagen fibers were significantly more numerous than COLIII fibers (*p* < 0.05). This composition underlies the physiological mechanical properties of intact tissue, providing high tensile strength with adequate extensibility. Intact tissues consisted of connective tissue structures consisting of the stratum reticulare of dermis and superficial fascia. Inflammation following foreign body implantation results in the formation of new connective tissue at the same site. Therefore, we suppose it is entirely appropriate to compare these tissues before and after implantation. Furthermore, comparing tissues for different implants was of paramount importance for the study.

Three months after implantation of synthetic materials, young connective tissue, as any granulation tissue, had formed in the peri-implant regions and contained numerous elastic fibers. In both implant groups, elastic fibers were significantly more abundant than in intact tissue before surgery (*p* < 0.05), with no significant difference between the polypropylene and biopolymer groups (*p* > 0.05).

By contrast, the volume density of COLI collagen in peri-implant tissue was significantly lower in both implant groups than in intact tissue (*p* < 0.05), and there was no significant difference in COLI between the polypropylene and biopolymer groups (*p* > 0.05). In the case of COLIII collagen, the groups differed significantly: the polypropylene group (Group 2) had a higher COLIII volume density than the biopolymer group (Group 3; *p* < 0.05). The pattern of COLI/COLIII ratios in intact and postoperative tissues was essentially reversed. In intact tissue, COLI predominated, whereas in peri-implant tissue from both groups COLIII predominated. This shift is biologically plausible, as type III collagen is actively produced during the proliferative and functional recovery phases of wound healing and is abundant in the walls of newly formed blood vessels, which are numerous in young connective tissue [11,14].

Despite the marked increase in elastic fibers in postoperative tissues in both groups, elastic fiber density remained significantly lower than total collagen density. In the polypropylene group, collagen and elastic fibers were oriented predominantly in parallel bundles surrounding the synthetic filaments, resembling dense, organized connective tissue. In the biopolymer group, fibers were more uniformly dispersed throughout the peri-implant region, resembling dense, irregular connective tissue.

On the basis of the qualitative and quantitative composition of stromal structures, the newly formed peri-implant tissue in both groups can be characterized as having good elasticity but reduced strength compared with intact tissue [12,14]. The connective tissue around the biopolymer is likely even weaker than that around polypropylene, owing to the more irregular fiber arrangement. In Group 2 (polypropylene), insufficient strength of the newly formed connective tissue is partially compensated by the integration of high-strength polypropylene filaments. In Group 3 (biopolymer), particularly after complete biotransformation of the material, this reinforcing component is absent. After 3 months of biopolymer implantation, the polymer matrix persists and may still contribute to mechanical support of the musculo-fascial wall. However, once the polymer has fully biotransformed, only young connective tissue with a relatively high elastic fiber content and a comparatively low collagen content remains at the former implant site. Whether this newly formed tissue can fully withstand the physiological mechanical loads normally borne by the musculo-fascial corset remains uncertain.

Implantation of a biodegradable polymer creates a time-dependent “implant-sur-rounding tissue” system within the body. Within this system, two opposing processes occur: progressive penetration of inflammatory infiltrate into the polymer matrix and a complex biotransformation of the material, including reduced bulk density due to degradation and cellular phagocytosis of polymer chains [21,22]. Biotransformation will depend on the time it takes for the polymer to break down (degrade) into small polymer chains suitable for cellular phagocytosis. The time it takes for biotransformation in tissues directly depends on the chemical and physical properties of the polymer and the time it takes for the small polymer chains to detach from the matrix. As a result, if the implant undergoes rapid resorption and is not replaced by sufficiently strong connective tissue, the newly formed tissue alone may not be able to function as an effective musculo-fascial corset for maintaining, for example, intra-abdominal pressure. This uncertainty can be resolved only empirically in future experiments, such as mechanical testing of explanted biopolymer constructs with surrounding tissue in experimental animals at various time points to assess stretchability and tensile strength. Such studies would help define the functional capacity of the “implant-surrounding tissue” system required for long-term correction of musculo-fascial defects.

On the basis of the present data, the suitability of this biopolymer for surgical correction of musculo-fascial wall failure cannot yet be confirmed. Further long-term studies are required. However, these findings suggest that the biopolymer could potentially be used, for example, as an adjunct in tension autoplasty of abdominal wall hernia defects to provide additional reinforcement alongside the patient’s own musculature, or for reconstruction of tissues where high elasticity, rather than maximal tensile strength, is the primary requirement.

## 5. Conclusions

Immunohistochemical analysis of stromal structures and morphometric evaluation of the cellular composition of connective tissue after surgery demonstrated broadly similar tissue responses to polypropylene and to an electrospun biopolymer composed of 65% polycaprolactone (PCL) and 35% polytrimethylene carbonate (PTMC). In both implant groups, young connective tissue formed with a predominance of type III (COLIII) collagen fibers, particularly around the polypropylene, in contrast to intact tissue in which type I (COLI) collagen predominated. Peri-implant tissues in both groups also contained more elastic fibers than intact tissue.

The fibroblastic response to the biopolymer lagged behind that to polypropylene by approximately 9 months, reflecting progressive biotransformation of the polymer matrix, but by 12 months after surgery the connective tissues in the two groups did not differ with respect to fibroblast and fibrocyte counts. Overall, the cellular composition and stromal organization of tissue surrounding the polypropylene and biopolymer implants did not show major qualitative or quantitative differences. However, the spatial arrangement of fibers differed, reflecting the distinct physical properties of the polymers. In peri-implant tissue from the polypropylene group, collagen and elastic fibers were oriented predominantly around the polypropylene filaments, creating a reinforcing effect. In peri-implant tissue from the biopolymer group, fibers were distributed more uniformly and diffusely. Dense, organized connective tissue formed around polypropylene by the second postoperative month, whereas dense, irregular connective tissue developed at the former biopolymer site by 9–12 months after complete biotransformation of the matrix.

On the basis of the qualitative and quantitative fiber composition, the newly formed connective tissue in both implant groups appears to have good elasticity but reduced strength compared with intact tissue. With polypropylene, this reduced strength is partially offset by the load-bearing mesh filaments. With the biopolymer, however, complete biotransformation within 12 months leaves only young connective tissue at the surgical site, without a persistent reinforcing scaffold. Accordingly, to assess the potential use of this biopolymer in hernia surgery, further studies are required, including late-stage immunohistochemical analysis of explanted tissues with detailed fiber architecture and experimental evaluation of the physical properties of peri-implant tissue, particularly extensibility and tensile strength.

## Figures and Tables

**Table 1:** The results of morphometry for different implanted materials in time, Mean ± SD (morphometry (%) in tissues, Group 1).

Implanted Material/Time		Fibrocytes	Fibroblasts
Polypropylene	2 weeks	23.4 ± 0.44	22 ± 1.2
Biopolymer	2 weeks	23.5 ± 1.6	21.4 ± 1.9
*p*		0.87	0.67
Polypropylene	1 month	35.6 ± 1.04	33.2 ± 1.1
Biopolymer	1 month	24.7 ± 0.8	22 ± 1.4
*p*		*p* < 0.05	*p* < 0.05
Polypropylene	2 months	37.4 ± 1.2	34.9 ± 0.8
Biopolymer	2 months	24.4 ± 1.5	22.2 ± 1.9
*p*		*p* < 0.05	*p* < 0.05
Polypropylene	3 months	38.3 ± 2.7	35.5 ± 3
Biopolymer	3 months	25.2 ± 2.5	21.6 ± 1.7
*p*		*p* < 0.05	*p* < 0.05
Polypropylene	6 months	39.1 ± 2.5	36.6 ± 2.4
Biopolymer	6 months	26.5 ± 3.4	23.2 ± 2
*p*		*p* < 0.05	*p* < 0.05
Polypropylene	9 months	40.8 ± 3.6	35.9 ± 3.6
Biopolymer	9 months	31.6 ± 2.4	28.9 ± 3.2
*p*		*p* < 0.05	*p* < 0.05
Polypropylene	12 months	39.7 ± 2.2	35.8 ± 3.5
Biopolymer	12 months	38.5 ± 2.3	36.8 ± 4.1
*p*		0.2	0.6

**Table 2:** The ratio of COLIII to COLI and the sum of COLI; COLIII to ELAST values in intact tissues and comparison groups.

Tissues	COLIII/COLI	(COLI + COLIII)/ELAST
Intact tissues	0.87	28
Group 2 (polypropylene)	3.25	6.76
Group 3 (biopolymer)	3.71	5.15

## Data Availability

The data that support the findings of this study are available from the corresponding author upon reasonable request.

## Abbreviations

The following abbreviations are used in this manuscript:

ASIA	Adjuvant-induced autoimmune/inflammatory syndrome
COLI	Collagen type I
COLIII	Collagen type III
ELASTs	Elastic fibers
PCL	Polycaprolactone
PTMC	Polytrimethylene carbonate
